# Protective Effect of Different Anti-Rabies Virus VHH Constructs against Rabies Disease in Mice

**DOI:** 10.1371/journal.pone.0109367

**Published:** 2014-10-27

**Authors:** Sanne Terryn, Aurélie Francart, Sophie Lamoral, Anna Hultberg, Heidi Rommelaere, Angela Wittelsberger, Filip Callewaert, Thomas Stohr, Kris Meerschaert, Ingrid Ottevaere, Catelijne Stortelers, Peter Vanlandschoot, Michael Kalai, Steven Van Gucht

**Affiliations:** 1 National Reference Centre of Rabies, Viral Diseases, Scientific Institute of Public Health (WIV-ISP), Brussels, Belgium; 2 Laboratory of Virology, Department of Virology, Parasitology and Immunology, Faculty of Veterinary Medicine, Ghent University, Merelbeke, Belgium; 3 Ablynx NV, Gent, Belgium; Thomas Jefferson University, United States of America

## Abstract

Rabies virus causes lethal brain infection in about 61000 people per year. Each year, tens of thousands of people receive anti-rabies prophylaxis with plasma-derived immunoglobulins and vaccine soon after exposure. Anti-rabies immunoglobulins are however expensive and have limited availability. VHH are the smallest antigen-binding functional fragments of camelid heavy chain antibodies, also called Nanobodies. The therapeutic potential of anti-rabies VHH was examined in a mouse model using intranasal challenge with a lethal dose of rabies virus. Anti-rabies VHH were administered directly into the brain or systemically, by intraperitoneal injection, 24 hours after virus challenge. Anti-rabies VHH were able to significantly prolong survival or even completely rescue mice from disease. The therapeutic effect depended on the dose, affinity and brain and plasma half-life of the VHH construct. Increasing the affinity by combining two VHH with a glycine-serine linker into bivalent or biparatopic constructs, increased the neutralizing potency to the picomolar range. Upon direct intracerebral administration, a dose as low as 33 µg of the biparatopic Rab-E8/H7 was still able to establish an anti-rabies effect. The effect of systemic treatment was significantly improved by increasing the half-life of Rab-E8/H7 through linkage with a third VHH targeted against albumin. Intraperitoneal treatment with 1.5 mg (2505 IU, 1 ml) of anti-albumin Rab-E8/H7 prolonged the median survival time from 9 to 15 days and completely rescued 43% of mice. For comparison, intraperitoneal treatment with the highest available dose of human anti-rabies immunoglobulins (65 mg, 111 IU, 1 ml) only prolonged survival by 2 days, without rescue. Overall, the therapeutic benefit seemed well correlated with the time of brain exposure and the plasma half-life of the used VHH construct. These results, together with the ease-of-production and superior thermal stability, render anti-rabies VHH into valuable candidates for development of alternative post exposure treatment drugs against rabies.

## Introduction

Rabies virus (*Familia Rhabdoviridae*, *Genus Lyssavirus*) is a model neurotropic RNA virus, which causes an aggressive and lethal infection in the brain of humans and mammals [1]. Once the virus enters peripheral nerves or neurons, the virus replicates quickly in the neuronal cytoplasm and progeny virus is transported through the neuronal network by crossing tight interneuronal synapses [2], [3].

Nanobodies (a trade-name by Ablynx) or VHH are the smallest functional portions (15 kDa) of heavy chain-only antibodies naturally occurring in *Camelidae*, and represent the antigen-binding variable domain. VHH consist of a single antigen-binding domain that does not require hydrophobic interaction with a light chain, leading to high solubility, physicochemical stability and high-yield production in *Escherichia coli* or yeast. The single domain nature and the small size of VHH allow easy formatting by genetic fusion into multimeric constructs with multiple specificities [4]–[6].

Previously, we developed a number of rabies virus-specific VHH directed against the rabies virus spike glycoprotein G [7]. *In vitro*, these VHH were fully able to neutralize the rabies virus infectivity in neuroblastoma and baby hamster kidney cells-21 (BHK-21) and could neutralize a wide spectrum of Lyssavirus species. The neutralizing potency increased massively when two VHH were combined with a glycine-serine linker into bivalent or biparatopic constructs [7].

Other research groups have developed antiviral VHH against a number of viruses [6]. For foot-and-mouth disease virus, rotavirus, respiratory syncytial virus and influenza virus, specific antiviral VHH were also tested in animal models. For these viruses, preventive treatment with VHH could delay or prevent disease upon challenge. In general, administration of VHH after infection had a limited effect on viral load or animal-to-animal transmission [8]–[11]. In the case of rotavirus, preventive treatment with VHH and continued administration until day 7 of infection was able to completely protect pigs from diarrhea, resulting in an asymptomatic infection and the development of a humoral immune response [11].

Anti-rabies antibodies are able to protect mice upon preventive administration and offer partial protection against disease and mortality upon early administration in a post exposure setting [12]–[14]. Antibody fragments, such as VHH or F(ab′)_2_, lack F_c_ domains, which render them incapable of exerting F_c_ effector functions, such as complement activation or interaction with F_c_ receptors on phagocytes. To what extent these effector functions contribute in control and clearance of infection, seems to depend on the virus [15]. In the case of influenza A virus, F_c_ effector function are not necessary for protection, whereas in the case of human immunodeficiency virus, the loss of F_c_γ-receptor binding function greatly increased the risk of infection upon pre-exposure treatment [16], [17].

F(ab′)_2_ fragments, obtained after pepsin digestion of whole antibodies, have reduced activity against rabies virus in mice [14]. Still, F(ab′)_2_ fragments derived from equine immunoglobulins are used in post exposure prophylaxis in humans as a cheaper substitute for human anti-rabies immunglobulins [18]. Recommended doses for equine F(ab′)_2_ (40 IU/kg) are two-fold higher than for human rabies immunoglobulins (20 IU/kg).

To date, little is known concerning the potential of VHH to neutralize rabies virus *in vivo* or to treat rabies virus infection. Viral receptors present *in vivo* are most likely different from the receptors responsible for virus uptake in cell lines [19]. Previously, Dietzschold *et al.* [20] found that the neutralizing potency of conventional antibodies determined in cells lines can differ substantially from their *in vivo* potency. Since VHH lack the F_c_ fragment of conventional antibodies, their *in vivo* antiviral activity might be compromised. A recent paper by the group of Boruah *et al.* [21] showed that pentameric constructs of anti-rabies VHH were able to partially neutralize rabies virus when co-injected with virus in the hindleg of mice. Constructs were composed of five homologous single domain antibody fragments fused to a coiled-coil peptide. The used dose was however relatively low (0.2–1.6 IU/ml). Also, the effect of treatment after virus challenge, as would occur under natural circumstances, was not examined.

In the current work we wanted to further explore the protective effect of anti-rabies VHH *in vitro* and *in vivo* using constructs with high antiviral potencies. Two homologous (bivalent) or heterologous (biparatopic) VHH were genetically fused with glycine-serine linkers to increase potency. Moreover, the *in vivo* circulating half-life of these constructs was extended by adding a third VHH targeted against albumin.

The aims of this study were to (1) compare the neutralizing potency of distinct monovalent, bivalent, biparatopic and half-life extended anti-rabies VHH both *in vitro* and *in vivo*, (2) assess the efficacy of anti-rabies VHH in delaying infection and disease, or even to rescue mice, by post exposure treatment after lethal intranasal virus challenge and (3) correlate the clinical outcome with the pharmacokinetic characteristics, including the total brain exposure, of anti-viral VHH.

## Results

### 1. Characterisation of the mouse rabies model

In order to test the *in vivo* efficacy of different anti-rabies VHH, a mouse model reflecting the neurological late stage of rabies disease was set-up and characterised. In a first series of experiments, disease symptoms and viral kinetics in the brain were assessed after intranasal inoculation of rabies virus. This route of inoculation allows the virus to directly access the brain via the olfactory epithelium, either through the olfactory nerve or the trigeminal nerve [22]. First disease signs appear at 7.1±0.67 days post inoculation (DPI) and severe neurological disease, requiring euthanasia, is observed at 8.3±0.88 days. Mortality is 100%.

**Figure 1:**
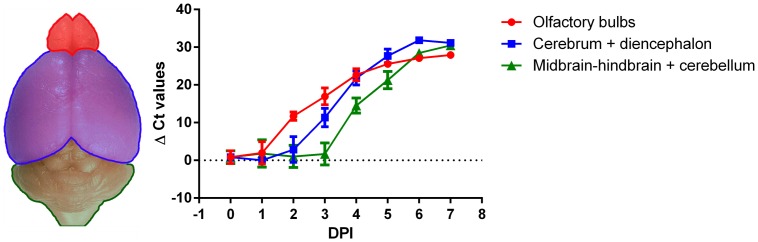
Virus spread in the mouse brain following intranasal rabies virus inoculation. The graph presents the profile of viral RNA in different parts of the brain (indicated in the left photo) upon intranasal inoculation of 10^2.5^ CCID_50_/mouse. Groups of mice (n = 7–10) were intranasally inoculated with rabies virus and sacrificed at various time points post inoculation (DPI). Viral loads were determined by qRT-PCR.

Virus spread through the brain over time was monitored by measuring the change in viral RNA load in the brain by quantitative real-time PCR (qRT-PCR) from 1 to 7 DPI, at which time clinical disease becomes obvious (Figure 1). Already at 1 DPI, virus can be detected in the olfactory bulbs (of 3/10 mice), with all mice being positive from 2 DPI onwards. The virus spreads from the frontal to the distal parts of the brain in a matter of days. In the cerebrum and diencephalon, viral RNA can be detected as soon as 2 DPI (in 4/7 mice) and from 3 DPI onwards in all mice. In the hindbrain and cerebellum, RNA can be detected as soon as 3 DPI (in 2/7 mice) and in all mice from 4 DPI onwards. Peak viral RNA levels (ΔCt≥25) are observed from 6 DPI onwards, which precedes the occurrence of severe neurological disease (score≥6) by 1 day. In conclusion, the intranasal inoculation of rabies virus provides an excellent infection model to study the efficacy of antiviral treatment in the brain. In contrast to intracerebral inoculation, it leaves the brain mechanically intact, and yields a highly reproducible brain infection and disease outcome with little variation in the median survival time.

### 2. Neutralizing potency of different anti-rabies VHH constructs *in vitro* and *in vivo*

#### 2.1. Co-administration of virus and VHH

In previous work, we described the generation of different anti-rabies glycoprotein G VHH constructs [7]. The virus-neutralization capacity of different monovalent, bivalent, biparatopic and half-life extended VHH constructs *in vitro* and in mice was compared (Table 1). Low doses of anti-rabies VHH (0.12 µg, 1 IU) were pre-incubated for 30 minutes at 37°C with the rabies virus, prior to administration to either BHK-21 cells or to different virus-receptive body compartments of the mouse (intranasal IN, intracerebral IC and intramuscular IM).

**Table 1:** Comparison of the neutralizing potency of different anti-rabies VHH constructs *in vitro* and *in vivo* following pre-incubation with rabies virus.

Compound			*In vitro* virus-neutralization (RFFIT assay in BHK-21 cells)		*In vivo* virus-neutralization: % mortality in mice (upon inoculation of a mix of 1 IU VHH and virus)		
			IU/µM	IC_50_ a nM	brain	nose	muscle
Phosphate-buffered saline			<0.5	>5881	100	100	50
Rabies monoclonal antibody RV1C5			193500	0.17	0	0	0
Irrelevant VHH	RSV-D3		<0.5	>5881	100	100	nd^b^
Anti-rabies VHH	Rab-C12		4.60	7.55	100	0	nd
	Rab-E6		2.54	13.66	57	0	nd
	Rab-E8		0.14	248.56	nd	nd	Nd
	Rab-E7		0.18	191.43	nd	nd	nd
	Rab-C12/C12	C12-15GS^c^-C12	8570	4.60	22	nd	nd
	Rab-E8/E8	E8-15GS-E8	9780	3.28	0	nd	nd
	Rab-H7/H7	H7-15GS-H7	15380	2.09	0	nd	nd
	Rab-E8/H7	E8-15GS-H7	230000	0.14	0	0	0
	Rab-E8/C12	E8-15GS-C12	8700	3.69	0	nd	nd
	Rab-E6/C12	E6-5GS-C12	10000	3.21	0	nd	nd
		E6-25GS-C12	6700	4.79	0	nd	nd
	Rab-E6/H7	E6-15GS-H7	93700	0.26	0	nd	nd
	HLE Rab-E8/H7	E8-15GS-H7-15GS-Alb	54388	0.91	nd	nd	nd

a IC_50_: 50% inhibitory concentration.

b nd: not determined.

c GS: glycine-serine.

All VHH constructs showed *in vitro* virus neutralization with IC_50_ values ranging from 0.1–15 nM whereas an irrelevant VHH construct was not active, confirming the specificity of the neutralization effect. The *in vitro* neutralization experiments showed that overall the potency of the VHH constructs increased significantly from monovalent to bivalent, and finally to the biparatopic constructs, with the latter having a comparable or higher potency as compared to the reference rabies monoclonal antibody.

*In vivo*, most bivalent and biparatopic constructs offered full protection in all the body compartments tested. Monovalent VHH could partially protect mice when the mix was administered intranasally, but not when administered directly into the brain. Remarkably, both monovalent and bivalent Rab-C12 VHH, while highly neutralizing *in vitro*, protected less well than other VHH *in vivo*. The biparatopic Rab-E8/H7 proved most potent *in vitro* and *in vivo*, and was thus selected for further *in vivo* studies. Addition of a third VHH (anti-human serum albumin) to Rab-E8/H7, to enable a longer circulating half-life, did not impact the *in vivo* neutralizing potency, despite of a limited reduction of the neutralizing potency *in vitro* (from 230000 IU/µM to 54388 IU/µM).

**Figure 2:**
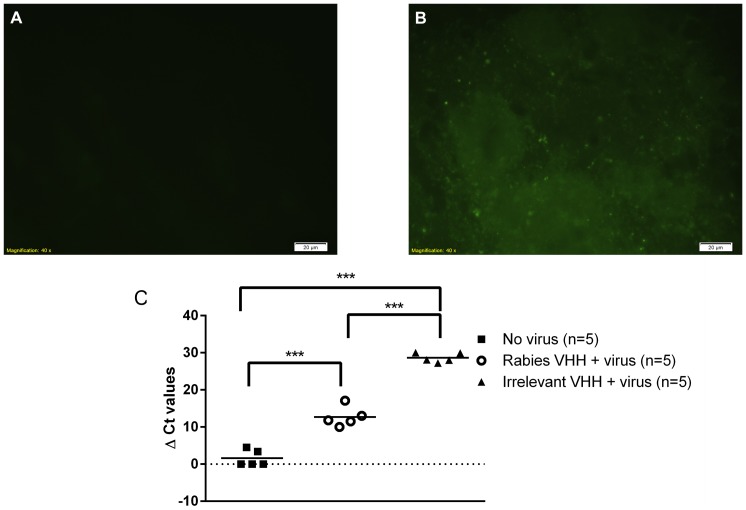
Co-administration of anti-rabies Rab-E8/H7 and virus directly in the brain efficiently inhibits virus infection. Mice were inoculated intracerebrally with a mix of rabies virus and 0.12 µg (1 IU) anti-rabies Rab-E8/H7 (A) or irrelevant VHH (B) and euthanized 7 days later. The anti-rabies VHH-treated mice were protected from disease, whereas all the mock-treated mice developed severe nervous disease. The pictures represent an immunofluorescence staining for viral nucleocapsid in the brain tissue. No viral antigens were visible in the brain of anti-rabies VHH-treated mice (A), whereas green fluorescent spots indicate the abundant spread of virus in the brain of mock-treated mice (B). The graph (C) presents the viral RNA load in the brains of different groups. Viral loads were significantly different between groups treated with Rab-E8/H7 and irrelevant control VHH, between Rab-E8/H7 and uninfected controls and between irrelevant control VHH and uninfected controls (*** p<0.01).

In addition, the neutralizing potency of Rab-E8/H7 was further investigated by determination of the viral load in the brain after co-administration of the VHH and the virus. Figure 2 shows that Rab-E8/H7 VHH efficiently inhibits the infectivity of rabies virus in the brain, as no viral antigen could be detected and only minimal levels of viral RNA at 7 DPI. The viral RNA load was significantly lower compared to that in the brain of control mice treated with an irrelevant VHH (p<0.001, t-test). In conclusion, most bivalent and biparatopic anti-rabies VHH could completely protect mice from disease upon inoculation of a pre-incubated mix of VHH and a lethal dose of rabies virus in different body compartments.

#### 2.2. Pre-exposure administration of anti-rabies VHH

In addition to the co-administration experiments, the most potent rabies VHH, Rab-E8/H7 was tested for efficacy upon preventive treatment. Pre-exposure treatment with a low dose of Rab E8/H7 (0.12 µg,1 IU) applied in the target organ (brain, IC) was followed by virus challenge in the nose 24 hours after VHH treatment. Complete protection was obtained against later IN virus challenge (p<0.01, Log Rank test, Bonferroni post-test). This indicates that a sufficiently high level of VHH remains present in the brain after one day, and diffuses from the site of injection, to neutralise the rabies virus upon brain entry via the nose (data not shown).

### 3. Post exposure treatment by direct intracerebral administration of Rab-E8/H7

#### 3.1. Administration of increasing doses at day one after virus inoculation

**Figure 3:**
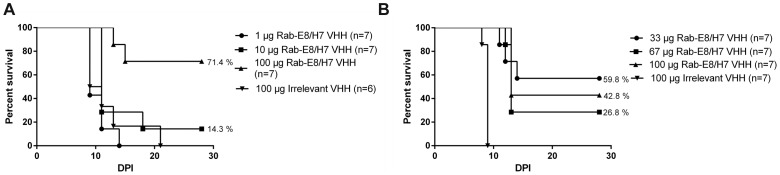
Dose-dependent efficacy of anti-rabies Rab-E8/H7 upon intracerebral post-exposure treatment at one day after intranasal virus inoculation. Two independent experiments were performed. In the first experiment (A), doses of 1, 10 and 100 µg Rab-E8/H7 were tested and in the second experiment (B), two additional doses of 33 µg and 67 µg were included. Significant protection was observed starting from a dose of 33 µg (p<0.01). One third to more than half of the mice that were treated at this or a higher dose survived the infection.

To determine the minimal effective dose to obtain protection against virus-induced mortality, mice were treated in two independent experiments with different doses of Rab-E8/H7 administered directly to the brain at one day after virus challenge. In a first experiment, mice were treated with 1 µg (4.63 IU), 10 µg (46.3 IU) or 100 µg (463 IU) at 24 hours after virus inoculation (Figure 3A). Treatment with 1 µg of Rab-E8/H7 gave no significant delay in the median survival time compared to animals treated with irrelevant VHH (9 days). A non-significant effect was observed at a dose of 10 µg Rab-E8/H7 and significant protection at a dose of 100 µg (p<0.01). To determine the effective dose more precisely, a second experiment was performed using two extra doses between 10 and 100 µg (Figure 3B). Significant protection was observed starting from a dose of 33 µg (p<0.01 for 33 µg, 67 µg and 100 µg, Log-Rank test, Bonferroni post-test). One third to more than half of the mice that were treated with a dose of 33 µg or higher survived the infection. There was no straightforward dose-response relationship in the second experiment, as the 33 µg dose performed better than the 67 and 100 µg doses. This inconsistency was probably due to experimental variation.

**Figure 4:**
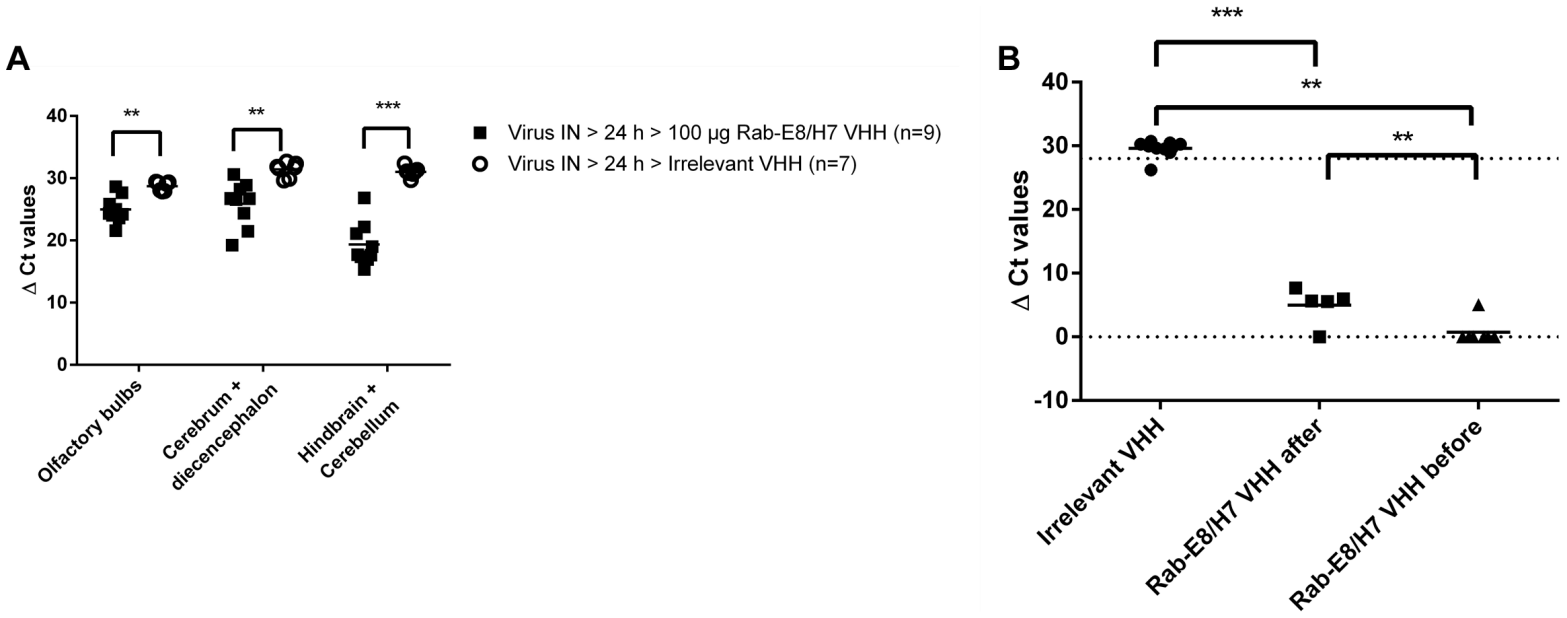
Viral RNA load in the brain after anti-rabies Rab-E8/H7 treatment. (A) Mice were treated with Rab-E8/H7 (100 µg) by intracerebral injection (IC) 24 hours after intranasal virus inoculation and sacrificed at 7 DPI to assess the viral RNA loads in different brain parts. Rab-E8/H7 VHH treatment significantly reduced the spread of the virus from the front to the posterior parts of the brain (t-test, ** p<0.01, *** p<0.0001). (B) Mice were treated with Rab-E8/H7 at 24 hours before (0.12 µg) or after (100 µg) intranasal virus inoculation. Control mice were mock-treated with irrelevant VHH before virus inoculation. Viral RNA loads were measured at 35 DPI in the brain of survivor mice. Four out of five survivor mice, treated after the virus inoculation, showed residual traces of viral RNA in the brain (ΔCt 5±2.9; *** p<0.0001). These mice had however never developed signs of disease. All mock-treated mice had to be euthanized at 7–9 DPI, because of serious disease, which coincided with high viral RNA loads in their brains (ΔCt≥28).

The antiviral effect of Rab-E8/H7 was further confirmed by monitoring the spread of the virus to the posterior parts of the brain by qRT-PCR (Figure 4). To this end, mice were treated 24 hours after intranasal virus inoculation by intracerebral injection of 100 µg Rab-E8/H7 (463 IU) or irrelevant VHH. Control mice were mock-treated with irrelevant VHH. Rab-E8/H7 VHH-treated mice had no symptoms at day 7, whereas mock-treated mice all presented serious nervous system disease symptoms at this time point. Figure 4A shows that Rab-E8/H7 treatment had significantly reduced the spread of the virus from the frontal to the posterior parts of the brain at day 7 after virus inoculation (t-test, ** p<0.01 for olfactory bulbs and cerebrum/diencephalon, *** p<0.0001 for hindbrain/cerebellum).

The long-term antiviral effect of intracerebrally injected Rab-E8/H7 (treatment before virus challenge: 0.12 µg, 1 IU or after: 100 µg, 463 IU) was also examined in survivor mice. Figure 4B shows the residual viral RNA load in the whole brain of survivor mice at the convalescent phase of infection (day 35). The survivor mice, which were treated either one day before or one day after virus inoculation, had only minimal amounts of viral RNA (ΔCt 5±2.9) in the brain at 35 DPI (*** p<0.0001), which demonstrates that they had successfully overcome the acute infection. Mice treated with an irrelevant VHH had to be euthanized around day 7–9 of infection and always contained high levels of viral RNA in the brain (ΔCt≥28).

#### 3.2. Administration at increasing time points after virus inoculation

**Figure 5:**
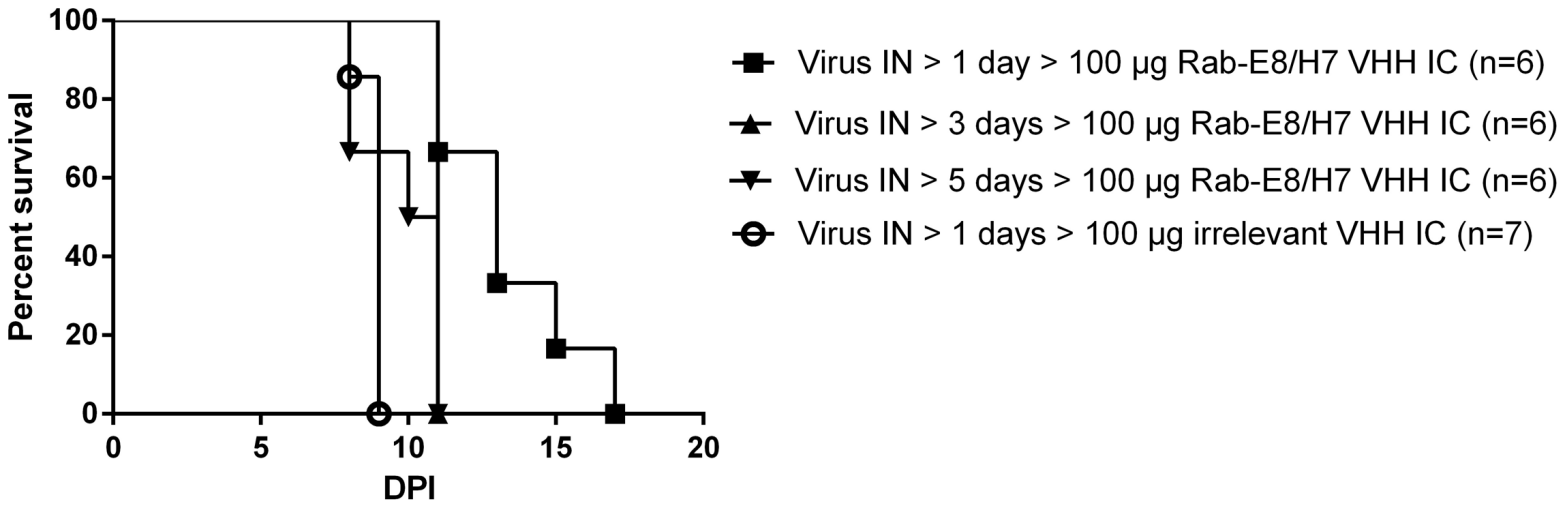
Post-exposure treatment by intracerebral injection at different time points of infection. Mice were treated with a single dose of 100 µg (463 IU) Rab-E8/H7 at increasing time points of infection. The protective effect of anti-rabies VHH diminished progressively when treatment was initiated at later stages of infection.

Given the protective nature of Rab-E8/H7 VHH treatment when given 1 day post-exposure, follow-up experiments were designed to elucidate the time-course of protection by Rab-E8/H7. Mice were treated with an intracerebral dose of 100 µg (463 IU) anti-rabies VHH at 1, 3 or 5 days after intranasal virus inoculation. The protective effect of Rab-E8/H7 VHH diminished progressively when treatment was initiated at later stages of infection (Figure 5). Median survival times were 13, 11 and 10.5 days upon starting Rab-E8/H7 VHH treatment at respectively day 1, 3 and 5 post virus inoculation, compared to a median survival time of 9 days in mock-treated mice. The prolongation of the median survival time was significant for treatment at day 1 or 3 (p<0.01, Log-Rank test, Bonferroni post-test), but not for treatment at day 5. Thus, 3 DPI was identified as the latest treatment time point to result in significant protection.

In two previous experiments (Figure 3A and B), 43–71% of mice treated IC with 100 µg Rab-E8/H7 at 1 DPI survived. In the third experiment (Figure 5), no mouse survived, albeit the median survival time was still significantly prolonged. This experimental variation may be explained because of small variations in the site of intracerebral injection.

### 4. Pharmacokinetic characteristics of anti-rabies VHH

**Figure 6:**
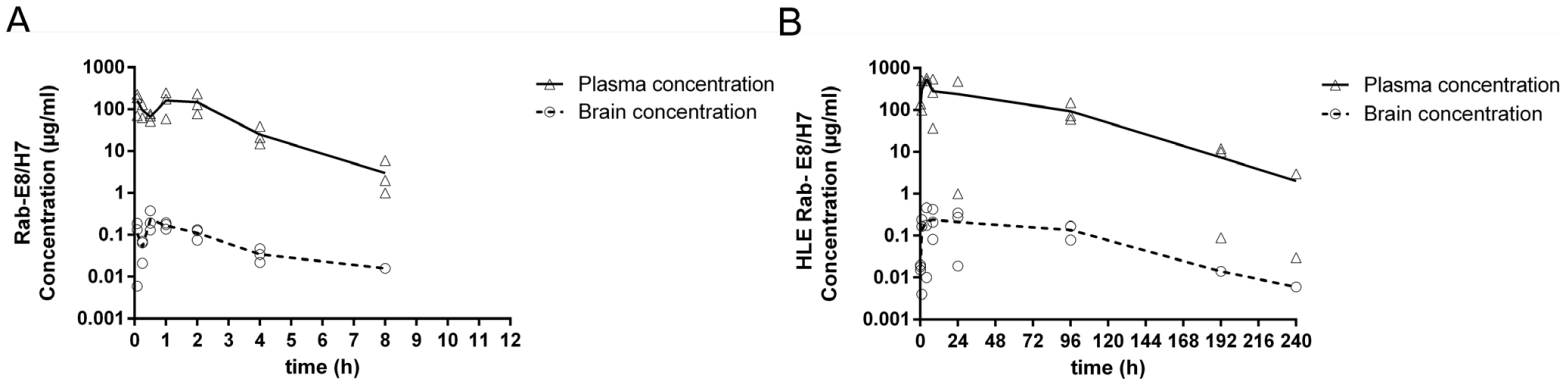
Mean brain and serum concentration of Rab-E8/H7 and HLE Rab-E8/H7. Individual brain (circles) and serum (triangles) concentrations and mean values (lines) of HLE Rab-E8/H7 (A) and Rab-E8/H7 (B) upon intraperitoneal injection of 5 mg HLE Rab-E8/H7 or 10 mg Rab-E8/H7.

**Figure 7:**
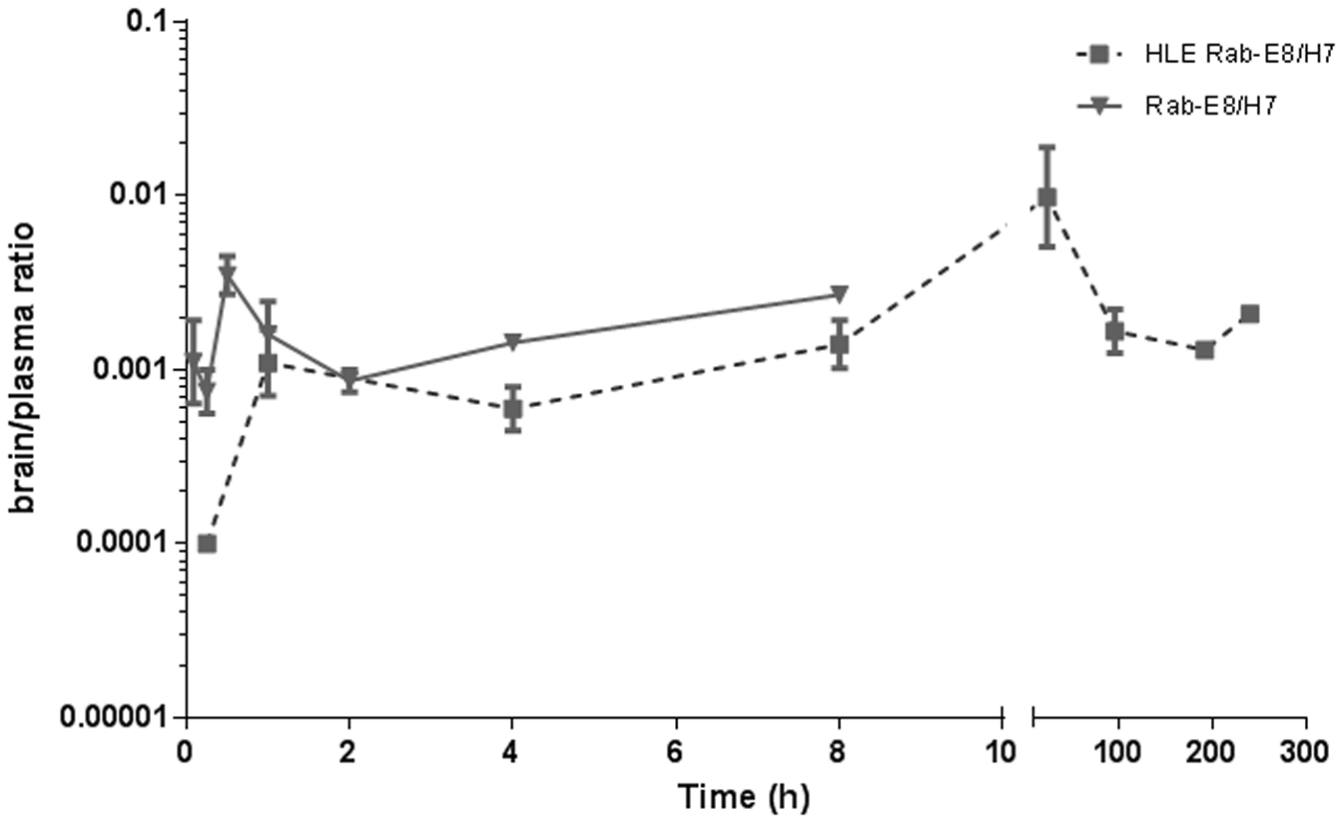
Mean brain/serum concentration ratio. Mean brain/serum concentration ratio over time for HLE Rab-E8/H7 and Rab-E8/H7 upon intraperitoneal injection of 5 mg HLE Rab-E8/H7 or 10 mg Rab-E8/H7 in mice.

A pharmacokinetic experiment was designed to determine the plasma and brain exposure following a single IP administration of Rab-E8/H7. In this experiment, Rab-E8/H7 was compared with a trivalent form of this VHH containing and extra anti-albumin VHH. Addition of this VHH is supposed to increase the half-life in circulation by binding to serum albumin. Both VHHs were administered in equimolar doses (5 mg Rab-E8/H7-ALB and 10 mg Rab-E8/H7). Serum versus time concentration profiles displayed a monophasic pharmacokinetic profile for both VHH constructs, but with an obvious rapid decline for Rab-E8/H7, which is likely explained by rapid renal filtration (Figure 6). Table 2 shows an overview of the mean calculated pharmacokinetic parameter estimates. Mean serum levels peaked at 0.08 h for Rab-E8/H7 and at 4 h for Rab-E8/H7-ALB, confirming the half-life extension (HLE) by addition of an anti-albumin VHH. We therefore refer to this VHH as HLE Rab-E8/H7. Maximum average brain levels were attained respectively at 0.5 h and 8 h after dose administration, revealing a fairly rapid influx into brain, which is indicative of a fast equilibrium between the blood and the brain. Due to the albumin-binding capacity of HLE Rab-E8/H7, a substantially higher systemic exposure (approximately hundredfold upon dose normalization) was attained for this half-life extended VHH. Similarly to what was seen in the blood, the brain was exposed to markedly higher VHH concentrations after dosing with the HLE Rab-E8/H7. Elimination of the VHH from the brain followed the same exponential disposition as in serum with no apparent accumulation in the brain. This was observed for both VHH constructs although slopes were steeper for the Rab-E8/H7 because of higher clearance rates. When differences in average systemic exposure were accounted for, both VHH constructs displayed a similar mean “area under the curve (AUC)”-based brain/serum ratio of approximately 0.1%, despite their differences in size and clearance rates. Also, mean brain/serum concentration ratios stayed fairly constant over time (Figure 7).

**Table 2:** Overview of average pharmacokinetic parameter values.

	Rab-E8/H7		HLE Rab-E8/H7	
Parameter	Serum	Brain tissue	Serum	Brain tissue
Cmax (µg/mL)	162	0.23	539	0.241
Tmax (h)	0.08	0.50	4	8
t_1/2_ (h)	1.16	2.06	30.5	41.7
AUC_infinity_ (h*µg/mL)	493	0.58	24613	26
AUC_infinity/_DOSE (h*µg/mL/µg)	0.0493	0.000058	4.93	0.0052

Values were estimated by non-compartmental analysis (sparse sampling, WinNonLin version 6.3, Phoenix Pharsight) for Rab-E8/H7 and HLE Rab-E8/H7 VHH in the serum and brain tissue. Brain concentrations were normalized for a theoretical brain weight of 0.5 g per animal and a density of 1 g/ml was assumed).

### 5. Post exposure treatment by systemic administration of anti-rabies VHH with or without half-life extension

In an effort to determine whether systemic administration of our anti-rabies VHH could also be protective, Rab E8/H7, both with and without the introduction of a half-life extension (HLE) module, was administered systemically one day after virus inoculation.

Mice that were treated by intraperitoneal injection with the non-HLE Rab-E8/H7 VHH at 24 hours after intranasal virus inoculation all developed disease and had to be euthanized (four independent experiments; n = 7 mice per treatment group). Nevertheless, administration of 10 mg (46300 IU) Rab-E8/H7, but not 2 or 5 mg (9260 and 23150 IU, respectively), consistently prolonged the median survival time by one day in all experiments (p<0.05, Log-Rank test, Bonferroni post-test) (data not shown).

**Figure 8:**
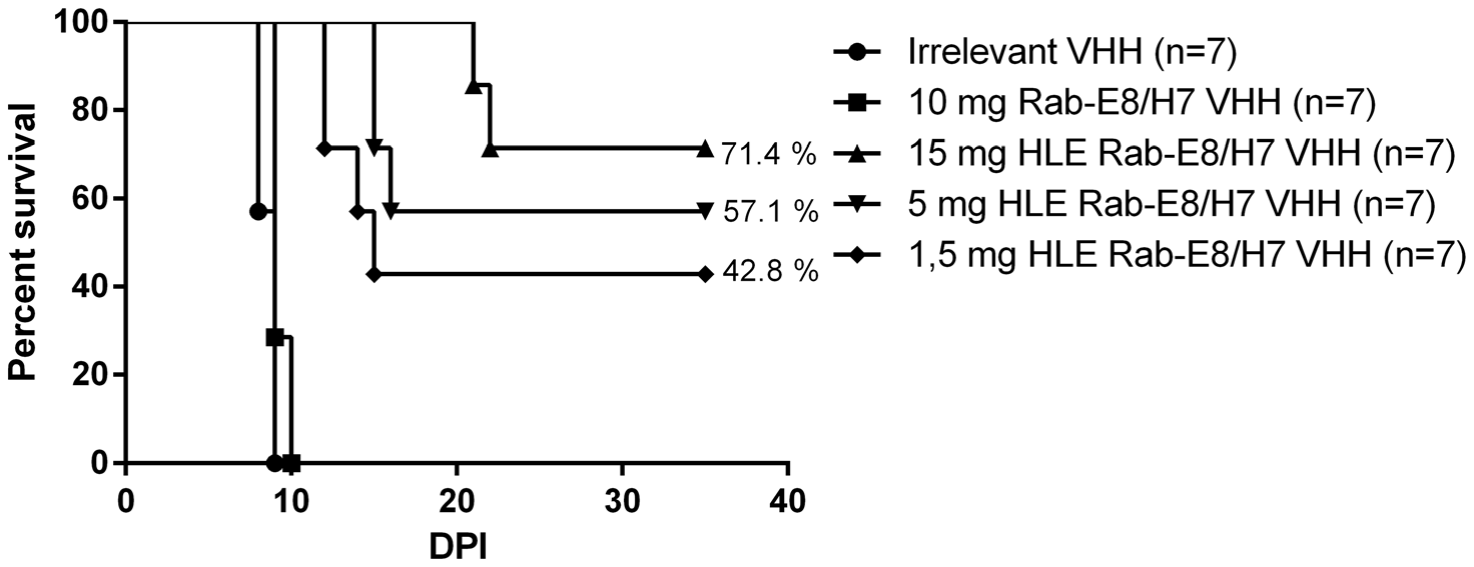
Post-exposure treatment with anti-rabies Rab-E8/H7 with or without half-life extension (HLE). Half-life extension was accomplished by adding a third anti-albumin VHH to Rab-E8/H7. Mice were treated intraperitoneally 24 hours after intranasal virus inoculation. The clinical effect of Rab-E8/H7 was significantly improved by the half-life extension. The median survival time was prolonged by six to more than 26 days (p<0.01), depending on the dose. More than 70% of the mice were completely protected against disease upon treatment with 15 mg HLE Rab-E8/H7.

Compared to conventional antibodies, VHH have a short plasma half-life (t_1/2_ Rab-E8/H7 = 1.16 h). To assess the efficacy of the HLE Rab-E8/H7 (t_1/2_ = 30.5 h), mice were treated by intraperitoneal injection with an equimolar dose (15 mg, 25050 IU) or lower doses (5 mg, 8350 IU and 1.5 mg, 2505 IU) of HLE Rab-E8/H7 at 24 hours after virus inoculation (Figure 8). Half-life extension of Rab-E8/H7 strongly enhanced the protective effect against rabies virus-induced mortality. In comparison to a one-day survival benefit seen with systemically delivered Rab-E8/H7, the introduction of half-life extension into the Rab-E8/H7 VHH improved median survival time by several weeks and resulted in a high percentage of animals being completed protected from lethal rabies challenge. This effect was significant (p<0.001) for all doses and dose-dependent on overall survival (3/7, 4/7 and 5/7 mice surviving, respectively) and median survival time (20 d, 26 d and >26 d, respectively).

Based on the pharmacokinetic studies, the enhanced efficacy and potency of the HLE Rab-E8/H7 can likely be explained by higher brain exposure secondary to prolonged plasma exposure, rather than to enhanced brain uptake.

**Figure 9:**
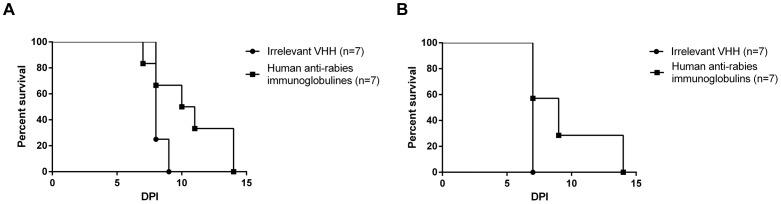
Post-exposure treatment with human anti-rabies immunoglobulins (Imogam). Mice were treated intraperitoneally with 65 mg (111 IU, 1 ml) of human rabies immunoglobulins at 24 hours after intranasal virus inoculation in two independent experiments (A and B). The median survival time was prolonged by 2 days, but all mice developed serious nervous disease, requiring euthanasia.

In order to benchmark the efficacy seen with the HLE Rab-E8/H7 VHH, similar experiments were conducted using commercially available human rabies immunoglobulins (RIG) purified from plasma of vaccinated human donors (Imogam, Sanofi Pasteur SA, Lyon, France). Intraperitoneal treatment with the maximal feasible dose of human RIG (1 ml intraperitoneally, 65 mg (111 IU)/mouse) was able to prolong the median survival time by two days (p<0.05) (Figure 9). All mice still developed serious neurological disease. The used dose of immunoglobulins was 308 times higher than the prescribed dose for humans (5550 IU/kg compared to 20 IU/kg) and represented the highest feasible dose which could be administered to mice, respecting a maximum IP injection volume of 1 ml.

## Discussion

In this study, the antiviral effect of different anti-rabies VHH constructs, targeted against the surface glycoprotein of the rabies virus, was examined in a brain infection model in mice. Monovalent, bivalent, biparatopic and half-life extended anti-rabies VHH were first compared *in vitro*. Then, a step-wise approach was used for extending the *in vitro* neutralisation results to *in vivo* neutralisation, starting with a pre-exposure setting and then testing the VHH in a prophylactic-therapeutic setting. Pre-exposure treatment and virus-VHH co-administration were primarily performed to proof the concept in a model with the highest chance on success.

Despite the absence of F_c_ effector functions and the small size, bivalent and biparatopic anti-rabies VHH are able to significantly prolong survival or even completely rescue mice from disease. The therapeutic effect depends on the time of treatment, dose, affinity, brain and plasma half-life of the VHH construct.

Increasing the affinity by combining two VHH with a glycine-serine linker into bivalent or biparatopic constructs, increased the *in vitro* neutralizing potency to the picomolar range. The potency of the different biparatopic constructs seemed to be higher than that of the monovalent or bivalent VHH, which has also been reported for other VHH directed against membrane receptors, such as CXCR4 and EGFR, where conformational changes play a role in the receptor activity [23], [24,24]. The inhibitory effect of the anti-rabies VHH might be due to steric hindrance, blocking the viral glycoprotein G and cell receptor interactions, or impairment of conformational changes in the G protein. Typically, biparatopic VHH are better in preventing conformational changes. However, the precise mechanism of VHH-mediated virus neutralisation remains unknown.

At day one of virus infection, direct intracerebral administration of the biparatopic Rab-E8/H7 at a dose as low as 33 µg was able to establish a significant anti-rabies effect. This is remarkable since the brain is extremely sensitive to infection and, in essence, only one infectious virus particle is sufficient to induce lethal infection in the absence of immunity [22].

Surprisingly, both monovalent and bivalent Rab-C12 VHH were highly neutralizing *in vitro*, but protected less well *in vivo*. Previously, we found that Rab-C12 recognizes a different epitope than Rab-E8 and Rab-H7 [7]. We did not map epitopes, but possibly the Rab-C12 epitope is less important for neutralisation *in vivo*. Correspondingly, Dietzschold *et al.* [20] already described that the neutralizing potency of antibodies can differ significantly *in vitro* and *in vivo*. Possibly, the virus uses different receptors for binding and uptake *in vitro* than *in vivo*.

Boruah *et al.* [21] reported that their pentavalent anti-rabies VHH constructs were able to partially (40–50%) protect mice against infection upon co-administration with virus in the hindleg. Our results confirm their obervations, albeit that both our monovalent and bivalent/biparatopic VHH constructs offered complete protection upon co-administration.

Obviously, when sufficient amounts of VHH are introduced in the brain at an early phase of infection (day 1), the further spread of virus slows down to such an extent that complete rescue of mice becomes feasible. Most likely, in survivor mice, the viral load never reached the critical threshold to induce disease. In our experience, a viral RNA load corresponding with a ΔCt of 28 or higher is associated with the appearance of severe nervous system disease. A delay in the build-up of virus, probably allows the immune response to kick in and clear out or control the virus infection. Indeed, upon post exposure treatment, survivor mice still harboured small amounts of residual virus in their brain (Figure 4A), but appeared in perfect health and had mounted neutralizing antibodies in their blood (data not shown).

The efficacy of post exposure treatment diminishes progressively when initiated at advanced stages of infection, varying from significant protection (treatment at day 1) to no protection (treatment at day 5) (Figure 5). At day 1, the spread of the infection is still limited to neurons of the olfactory bulbs (Figure 1), which agrees with previous studies [22], [25]. At this early stage, treatment can still prevent or delay spread to the rest of the brain. At later stages, the virus has spread over larger parts of the brain. We assume that anti-rabies VHH are able to intercept intercellular virus spread, but can not diminish or clear out intracellular virus, which limits the effect of VHH treatment at more advanced stages of infection.

Compared to direct intracerebral treatment (33 µg), much higher doses (10 mg) are needed to delay disease or protect mice upon systemic treatment. The relative weaker performance of systemic treatment can easily be explained by the fact that only a small fraction of Rab-E8/H7 eventually reaches the brain.

Our pharmacokinetic data on systemic administration support that the therapeutic benefit depends on the time of brain exposure and the plasma half-life of the used VHH construct. Indeed, half-life extension (HLE) of Rab-E8/H7, by adding a third VHH targeting serum albumin, considerably improved the therapeutic effect. Systemic administration of HLE Rab-E8/H7 resulted in a prolonged survival of at least six days and complete protection from disease in part of the mice (43–71%) in a dose-dependent manner (Figure 8). Upon intraperitoneal administration, the brain level of Rab-E8/H7 peaked 0.5 h after injection and Rab-E8/H7 was almost completely cleared from the brain within one day, whereas HLE Rab-E8/H7 peaked 8 h after injection and remained clearly detectable for ten days. This agrees with the delay in disease onset with one day for Rab-E8/H7 and with six days for HLE RabE8/H7.

The enhanced exposure in the brain of HLE Rab-E8/H7 seems mainly due to the prolonged retention in the systemic circulation. We found no evidence for active blood-brain barrier crossing with the tested (HLE) anti-rabies VHH, as the brain levels followed the systemic exposure. The brain levels of (HLE) VHH represented 0.1% the plasma levels suggesting a limited diffusion of anti-rabies VHH to the brain, corresponding with distribution data also described for antibodies [26], [27]. It has been reported that certain VHH, directed against glial fibrillary acidic protein, that contain many positively charged residues, show enhanced crossing of the blood brain barrier, presumably via an adsorption-mediated uptake mechanism [28]. In this respect, it should be noted that the VHH building blocks of Rab-E8/H7 each have isoelectric point (pI) values around 7 and hence are neutrally charged. Our pharmacokinetic results do not indicate that the rabies VHH constructs used in our study can cross the blood-brain barrier to a higher extent than reported for antibodies, despite their smaller size. Modification of VHH by adding blood-brain barrier targeting peptides might be an interesting future strategy to increase the therapeutic potential [29].

For comparison, intraperitoneal treatment with the highest available dose of human rabies immunoglobulins (RIG, Imogam, 65 mg, 111 IU, 1 ml) only prolonged survival by two days, without rescue (Figure 9). The applied dose of RIG corresponded to the highest volume of Imogam which could be administered to mice and was about 308 times higher than the dose used in humans. Treatment with HLE Rab-E8/H7 (1.5 mg, 2505 IU/mouse) was more effective than treatment with RIG, probably due to the higher neutralizing dose achieved with HLE Rab-E8/H7. Still, it should be noted that HLE Rab-E8/H7 has a shorter plasma half-life (30.5 h) than human immunoglobulins (8 days) in mice [30].

The current work is presented as a proof-of-concept study, without direct translation for clinical development. Still, we think that HLE anti-rabies VHH have the potential to be used as an alternative to RIG or monoclonal antibodies to provide passive immunity after risk exposure. Of course, further validation in animal models and trials in humans are needed to enforce this, as the current study design and animal model does not allow validation of VHH for post exposure prophylaxis in humans.

Rabies virus-exposed patients are currently treated with RIG and vaccine soon after exposure [31]. Patients receive RIG and vaccine on day 0 and additional vaccine shots in the following weeks. The rationale is that patients need continued protection by anti-rabies antibodies starting as soon as possible after exposure. First protection is offered by passively acquired antibodies (RIG), which are then gradually replaced by vaccine-induced antibodies. Active antibodies are first mounted between day 7 and 14 [32], so passively acquired antibodies need to bridge the immunity gap between day 0 and 7–14. We believe that anti-albumin VHH can easily be dosed to provide sufficient passive immunity for the first 7–14 days after exposure in humans. Indeed, the albumin-binding VHH used in our study is known to have a longer plasma half-life (10–20 days) in humans than in mice, which bodes well for the potential use of the anti-rabies HLE Rab-E8/H7 VHH in humans [33]. Importantly, the systemic levels of RIG obtained at the recommended dose (20 IU/ml) remain remarkably low (0.01–0.50 IU/ml) in humans [32], which is why the WHO recommends to infiltrate RIG as much as possible locally in or around the infected wound. In our experience, most RIG are administered systemically, not at least because the bite wound is often localized in a small body extremity, such as the nose or a finger, which does not allow injection of a large volume (risk of compartment syndrome). Interestingly, VHH can easily be concentrated to a small volume which is better suited for injection in small body parts.

These results provide evidence for the possible use of anti-rabies VHH as valuable candidates for the development of alternative post exposure treatment drugs for rabies. The ease of production and high thermal stability of VHH are important advantages over the currently used anti-rabies immunoglobulins. A further increase of the half-life [34], blood-brain barrier crossing properties [28] and/or brain delivery of systemically administered anti-rabies VHH might further improve the therapeutic applicability of anti-rabies virus VHH.

## Materials and Methods

### VHH and antibody

VHH directed against the rabies virus glycoprotein G were generated previously [7]. Briefly, llamas were vaccinated with the inactivated rabies Human Diploid Cell Vaccine (HDCV, Sanofi, France) and RNA was extracted from peripheral blood lymphocytes. VHH genes were amplified from a cDNA library. Anti-rabies virus VHH were selected by panning phage libraries on plates coated with the native G protein. For the generation of multivalent VHH constructs, monovalent VHH were genetically fused into dimeric VHH constructs using flexible glycine-serine (GS) linkers [8]. Bivalent VHH contained two identical VHH monomer clones, whereas biparatopic VHH contained two different VHH clones. RSV117, a bivalent respiratory syncytial virus (RSV)-specific VHH, was used as a negative irrelevant control VHH. The Rab-E8/H7 VHH was genetically fused with a VHH directed against human serum albumin to extend the half-life using a GS linker, resulting in HLE-Rab-E8/H7. The activity of the resulting HLE Rab-E8/H7 VHH was tested in the virus-neutralization assay to confirm that the fusion did not impact the rabies neutralization. For initial characterization, all VHH were produced with C-terminal cMyc-His6 tags in *Eschericha coli*. For subsequent *in vivo* experiments Rab-E8/H7 and HLE Rab-E8/H7 were recloned to *Pichia pastoris* expression vectors for production in X-33 strain as tag-less proteins. All VHH were purified to endotoxin levels <5 EU/mg.

Human rabies immunoglobulins (HRIG) (Imogam, Sanofi Pasteur SA, Lyon, France) are gammaglobulins purified from plasma of vaccinated human donors.

### Virus

Challenge Virus Standard (CVS)-11 is a virulent classical rabies virus obtained from the American Type Culture Collection (ATCC reference VR959) and was grown in baby hamster kidney (BHK)-21 cells (Deutsche Sammlung von Mikroorganismen und Zellkulturen GmbH, Braunschweig, Germany). For virus inoculation in mice, a dose of 10^2.5^ 50% cell culture infectious doses (CCID_50_) of CVS-11 was used.

### Virus-neutralization in vitro

The virus-neutralizing potency was titrated with the rapid fluorescent focus inhibition test (RFFIT) according to the Manual of Diagnostic Tests and Vaccines for Terrestrial Animals (Office International des Epizooties, 2008). Briefly, a standard dose of virus was pre-incubated with serially diluted VHH/antibody for 90 min at 37°C. BHK-21 cells were then added to the mix and co-incubated for 24 hours. Infected BHK-21 cells were stained with fluorescent anti-nucleocapsid antibody and foci of infected cells were counted under the fluorescence microscope. The dilution that yielded 50% inhibition of infected foci was determined. The neutralizing potency is expressed in international units (IU)/ml in reference to “The Second International Standard for Anti-Rabies Immunoglobulin”, purchased from the United Kingdom National Institute for Biological Standards and Control.

### Rabies ELISA

The Platelia TM Rabies II Kit from Bio-Rad (Cat number: 3551180) was used for assessing functional activity of the different anti-rabies VHH constructs. A dilution series (in R6 buffer provided with the kit) of the anti-rabies VHH was incubated on the wells precoated with rabies virus glycoprotein for 1 h at 37°C. After washing, bound VHH was incubated for 1 h at 37°C with anti-VHH antibodies (in house produced, R345 rabbit anti-VHH polyclonal antibodies, 1/2500) followed by an incubation with an anti-rabbit-HRP antibody (BETHYL, Cat A120-201P, 1/1000) for 1 h at 37°C. TMB (es(HS)TMB, Pierce) substrate was added and incubated in the dark at room temperature, the reaction was stopped after 30 min by addition of 1 M HCl. Read-out was done at 450–620 nm.

### Mouse inoculation experiments

Six-to-eight weeks old female Swiss outbred mice (Charles River, France) were used in all experimental procedures. The experimental procedures were approved by the local ethical committee of the institute (advice n° 070515-05). Mice were kept in filter top cages, water and feed were provided *ad libitum* and exposed to a natural day/night light cycle. The intracranial (IC), intranasal (IN) and intraperitoneal (IP) inoculation procedures are described in detail by Rosseels *et al.* [35]. For IC, IN and IP injection volumes of respectively 20, 25 and 1000 µl were used.

The intranasal inoculation of rabies virus is an excellent technique to study antiviral treatment in the brain, since it leaves the brain mechanically intact, in contrast to intracerebral inoculation, and yields a highly reproducible brain infection and disease outcome with little variation in the median survival time [35]. This inoculation route has been used before for the evaluation of post exposure prophylaxis of rabies in mice [14].

Prior to inoculation with virus or VHH, mice were briefly anesthetized using isoflurane gas (IsoFlo, Abbott laboratories Ltd., Queenborough, Kent, United Kingdom).

### Determination of viral kinetics in the brain

The viral RNA load was determined using real-time reverse transcriptase polymerase chain reaction (RT-qPCR), as described by Suin *et al.* [36], in the whole brain, or in the olfactory bulbs, mid (cerebrum and diencephalon) and anterior (hindbrain and cerebellum) parts of the brain. Primers recognize the nucleocapsid region of genomic RNA [36]. Previously, Suin *et al.* [36] found a good correlation between viral RNA load and infectious virus titer in the brain. Brain samples were homogenised using a tissue homogenizer (Bullet Blender, Next Advance, New York, USA) and 5 mm stainless steel beads in 350–1000 µl lysis buffer (RLT buffer, as supplied with Qiagen RNeasy kit, with 1% β-mercaptoethanol). Total RNA was extracted using the Qiagen RNeasy kit (Qiagen, Hilden, Germany), according to manufacturer's instructions. RNA was quantified with the NanoVue spectrophotometer (GE Healthcare, Bucks, UK). A CVS-11 standard curve was established for each plate based on serial dilutions of a well-defined virus stock. Ribosomal 18S or GAPDH were used as reference genes for standardization. Delta cycle thresholds (ΔCt) values were calculated using the following formula: ΔCt = Ct_ref_ - Ct, with Ct_ref_ equal to 45, which is the number of cycles of this qPCR program.

The fluorescent antigen test (FAT) was performed according to the Manual of Diagnostic Tests and Vaccines for Terrestrial Animals (Office International des Epizooties, 2008). Brain smears were fixed with 75% acetone for 30 min at −20° and incubated with FITC-coupled anti-nucleocapsid rabbit antibody for 30 min at 37°C.

### Pharmacokinetic study

The plasma and brain disposition upon systemic administration of the Rab-E8/H7 VHH and its albumin-binding counterpart was investigated in a pharmacokinetic study. To this end, 48 animals were treated with a single intraperitoneal injection of 10 mg Rab-E8/H7 or 5 mg HLE Rab-E8/H7. Three mice were sacrificed per sampling time point. Immediately before euthanasia, each mice received a transcardial perfusion with phosphate-buffered saline (PBS) [37]. Briefly, mice were injected intraperitoneally with a mixture of xylazine (Rompun 2%, Bayer Healthcare, Kiel, Germany, 9.9 mg/kg) and ketamine (Ceva, Brussels, Belgium, 100 mg/kg) to induce deep terminal anaesthesia. Upon the disappearance of the eye lid and motor reflexes, the thorax was opened to expose the heart. Twenty ml of PBS of 37°C was injected directly into the left ventricle of the heart at a steady perfusion rate of 10 ml/min. An incision was made in the right heart chamber to drain out the blood from the circulatory system. After perfusion, both brain halves were collected and snap frozen in dry ice.

One brain halve was homogenized in ice cold PBS supplemented with 1 mM phenylmethanesulfonylfluoride (PMSF) and protease inhibitor cocktail (VWR International) using 5 mm stainless steel beads in a tissue homogenizer (Bullet Blender, Next Advance, New York, USA). For homogenization of the brain samples a fixed volume of 1.25 ml was used (approximately a 1∶10 ratio of tissue: lysis buffer). Homogenates were subsequently centrifuged for 20 min at 13000 g and supernatant was transferred to a new tube and stored at −80°C until quantification. The total amount of protein present in the homogenates was determined using the Bradford method. For the calibration curve a dilution series of BSA was used ranging from 1.0 to 0.063 mg/ml. 20 µl of standard and samples (1/30 dilution) was mixed with 300 µl Bradford Ultra Reagent (Expedeon; CatBFU1L). Absorbance at 595 nm was measured and concentration of samples was interpolated from the standard curve.

For ELISA analysis, total brain amounts were calculated and were normalized for a theoretical brain weight of 0.5 g *(source* www.mbl.org/atlas170 *)* per mouse brain. A density of 1 g/ml was assumed to calculate VHH brain concentration [38]. Undiluted brain lysate or plasma samples that resulted in signals below the assay's limit of quantification (0.1 ng/ml) were considered as missing. Pharmacokinetic parameters were estimated by non-compartmental analysis (Plasma Model Type 200, sparse sampling) using WinNonLin software version 6.3 (Phoenix Pharsight, Mountain View, CA).

The average maximum concentration in plasma (C_max_) and corresponding mean time (t_max_) were directly derived from the plasma concentration-time profiles. The area under the plasma concentration-time curve from the time of dosing to the time of the last measurable concentration (AUC(0-t)) was calculated by the linear trapezoidal rule and extrapolated to infinity (AUC_inf_) as AUC(0-t)+Ct/λz, in which λz, the first order rate constant associated with the terminal elimination phase, was estimated by linear regression of time versus log concentration. The half-life (t_1/2_) of the terminal elimination phase was calculated as ln(2)/λz.

### Quantification of anti-rabies VHH in plasma and brain homogenates

To quantify the amount of anti-rabies VHH in the homogenized brain tissues and plasma, the samples were tested in a virus-neutralization assay (RFFIT) or in an ELISA using the Platelia TM Rabies II Kit from Bio-Rad (both as described above). For the standard curve, a serial 1.7 dilution series of the anti-rabies VHH ranging from 250 to 0.05 ng/ml in 20% brain matrix was used. Brain samples were measured at 1/5 dilutions, plasma samples at dilutions ranging from 1/25 to 1/900, all in duplicate. Samples (50 µl/well) were transferred to the plate provided by the kit. The linear range of the standard curve was determined using 4PL analysis (Graphpad Prism), which ranged from 0.1 to 10 ng/ml. The assay's range and accuracy was confirmed with spiked VHH controls of 1, 2 and 5 ng/ml. Concentrations of unknown samples were determined by interpolation from the standard curve. For the plasma samples, the reported concentration was derived from averaging the values of the different dilutions.

### Clinical follow-up

Mice were observed daily for signs of disease throughout the experiment until maximum 35 days after virus inoculation. Signs of disease were evaluated as follows: weight loss (0 = absent, 1 = visible), depression (0 = absent, 1 = lower (re)activity), hunched back (0 = absent, 1 = present), motoric incoordination (0 = absent, 1 = present), rough hair coat (0 = absent, 1 = present), paralysis of the hind legs (0 = absent, 1 = present) and conjunctivitis (0 = absent, 1 = present). The cumulative daily clinical score per mouse was calculated as the sum of the scores for each parameter. Disease progression was represented by plotting the cumulative daily score in function of the days post inoculation (DPI). The cumulative daily score per mouse ranged from 0 (no disease) to 7 (severe nervous disease). In our experience, mice with a disease score of 6 or more die within one day [35]. Therefore, mice were euthanized by cervical dislocation when they reached a disease score of ≥6. Results were expressed as Kaplan-Meier survival curves. GraphPad Prism was used for statistical analyses of *in vivo* data. Differences in survival times were tested using the Log-Rank test with a Bonferroni post-test, differences in ΔCt values were tested using a student t-test after normalization to the house-keeping gene.

### Ethics statement

The animal experiments were approved by the Local Ethical Committee of the Scientific Institute of Public Health (WIV-ISP) and the Veterinary and Agrochemical Research Centre (CODA-CERVA, Brussels, Blreference numbers EC 070515-04 (llama experiments), EC 070515-05 and EC 120125-01 (mouse experiments). The ethical committee is recognized by the Belgian federal authority and complies with the Belgian Federal Law of the 14^th^ of August 1986 and the 27^th^ of December 2012. Animal experiments were conducted according to the guidelines of Directive 86/609/EEC and Directive 2010/63/EU of the European Parliament and of the Council of 22^nd^ of September 2010 on the protection of animals used for scientific purposes.
